# A Computational Framework for 3D Mechanical Modeling of Plant Morphogenesis with Cellular Resolution

**DOI:** 10.1371/journal.pcbi.1003950

**Published:** 2015-01-08

**Authors:** Frédéric Boudon, Jérôme Chopard, Olivier Ali, Benjamin Gilles, Olivier Hamant, Arezki Boudaoud, Jan Traas, Christophe Godin

**Affiliations:** 1 Virtual Plants Inria team, UMR AGAP, CIRAD, INRIA, INRA, Montpellier, France; 2 Laboratoire de Reproduction et Développement des Plantes, Université de Lyon 1, ENS-Lyon, INRA, CNRS, Lyon, France; 3 Laboratoire d'Informatique, de Robotique et de Microélectronique de Montpellier, Université Montpellier 2, CNRS, Montpellier, France; Princeton University, United States of America

## Abstract

The link between genetic regulation and the definition of form and size during morphogenesis remains largely an open question in both plant and animal biology. This is partially due to the complexity of the process, involving extensive molecular networks, multiple feedbacks between different scales of organization and physical forces operating at multiple levels. Here we present a conceptual and modeling framework aimed at generating an integrated understanding of morphogenesis in plants. This framework is based on the biophysical properties of plant cells, which are under high internal turgor pressure, and are prevented from bursting because of the presence of a rigid cell wall. To control cell growth, the underlying molecular networks must interfere locally with the elastic and/or plastic extensibility of this cell wall. We present a model in the form of a three dimensional (3D) virtual tissue, where growth depends on the local modulation of wall mechanical properties and turgor pressure. The model shows how forces generated by turgor-pressure can act both cell autonomously and non-cell autonomously to drive growth in different directions. We use simulations to explore lateral organ formation at the shoot apical meristem. Although different scenarios lead to similar shape changes, they are not equivalent and lead to different, testable predictions regarding the mechanical and geometrical properties of the growing lateral organs. Using flower development as an example, we further show how a limited number of gene activities can explain the complex shape changes that accompany organ outgrowth.

## Author Summary

In recent years, much research in molecular and developmental biology has been devoted to unravelling the mechanisms that govern the development of living systems. This includes the identification of key molecular networks that control shape formation and their response to hormonal regulation. However, a key challenge now is to understand how these signals, which arise at cellular scale, are physically translated into growth at organ scale, and how these shape changes feed back into molecular regulation systems. To address this question, we developed a computational framework to model the mechanics of 3D tissues during growth at cellular resolution. In our approach, gene regulation is related to tissue mechanical properties through a constitutive tensorial growth equation. Our computational system makes it possible to integrate this equation in both space and time over the growing multicellular structure in close to interactive time. We demonstrate the interest of such a framework to study morphogenesis by constructing a model of flower development, showing how regulation of regional identities can, by dynamically modulating the mechanical properties of cells, lead to realistic shape development.

## Introduction

The control of form and size is a central issue in developmental biology. It is commonly accepted that genetic regulation is at the basis of morphogenesis. However, while molecular genetics has provided an important number of actors required for morphogenetic events, the link between these regulators and global shape control remains largely an open question in both plant and animal biology. Furthermore, the contribution of multicellularity in shape changes and growth remains poorly explored. In fact, certain species exhibit complex shapes while being composed of only one giant and multinucleated cell (see S1 Fig.). This raises the question of the exact contribution of the presence of neighboring cells in the growth of a given cell, within a tissue. So far, this issue has mainly been addressed from a signaling point of view (e.g. diffusion of morphogens [1], mechanical feedbacks [2], [3]), but the role of multicellularity in the biophysics of growth remains to be formalized.

Here we consider this issue in plants. Plant cells are under high internal turgor pressure and it is only the presence of a rigid exoskeleton that prevents them from bursting. This exoskeleton, the cell wall, is composed of a dense network of cellulose microfibrils that are cross-linked to each other by a network of polysaccharides. Sachs, as early as in 1882 (reviewed in e.g. [4], [5]) discovered that cell expansion can only take place as long as the cells are under pressure, which has led to the concept of turgor-driven cell growth. It is now widely accepted that this involves the irreversible (plastic) yielding of the cell wall to this pressure, e.g. [6].

Based on this general concept of wall yielding a now widely accepted general scenario was proposed by Lockhart to describe the growth of an isolated cell [7]. This scenario can be summarized as follows. In a non-growing isolated cell, the internal pressure is counterbalanced by the tension in the cell wall. If this pressure further increases and reaches a certain threshold, the load bearing parts of the cell wall yield. Lockhart [7] proposed to model this viscoplastic process with a simple relationship between 2 key variables, the relative rate of growth of the cell volume 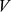, and the cell turgor pressure 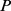: if the pressure is greater than a fixed threshold 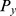 and the flow of water is not a limiting factor, then the cell yields and the rate of growth is proportional to the excess of turgor pressure:
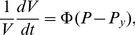
(1)


where 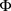 denotes the extensibility of the cell, i.e. its ability to grow under a given pressure, the inverse of its viscosity. If the turgor pressure does not reach the yield threshold 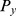, no growth is achieved and the cell deformation is entirely elastic (reversible). Above 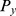, the cell deformation becomes plastic (irreversible). The potential decrease in pressure due to cell growth is continuously compensated by further water uptake, thus keeping the wall under continuous tension [4]. In other words, in a single cell system, growth can be entirely described in terms of the variations of the internal turgor pressure and of the mechanical properties of the cell wall.

The initial formulation of Lockhart and subsequent models did not account for cell geometry and anisotropic properties of wall material. Recently, Dumais and coworkers applied Lockhart's model of cell growth to cell walls, and extended it to account for wall anisotropic properties [8]. They introduced a model of tip-growing cells (e.g. root hairs) that combines two key processes of cell growth, namely the deposition of material on cell walls and the mechanical deformation of the cell wall due to stresses resulting from the cell's inner turgor pressure. Interestingly, the authors show that the Lockhart growth equation can be simply extended in 3 dimensions to take into account wall anisotropy. This leads to 3 equations (instead of one) that express how the rate of deformation in the 3 directions of space are affected by mechanical anisotropy in the cell walls [8]. With the help of this model, the authors could analyze the dynamics of a tip growing cell, and how the visco-elastic properties of cell walls may impact its shape in steady or non-steady regimes.

**Figure 1:**
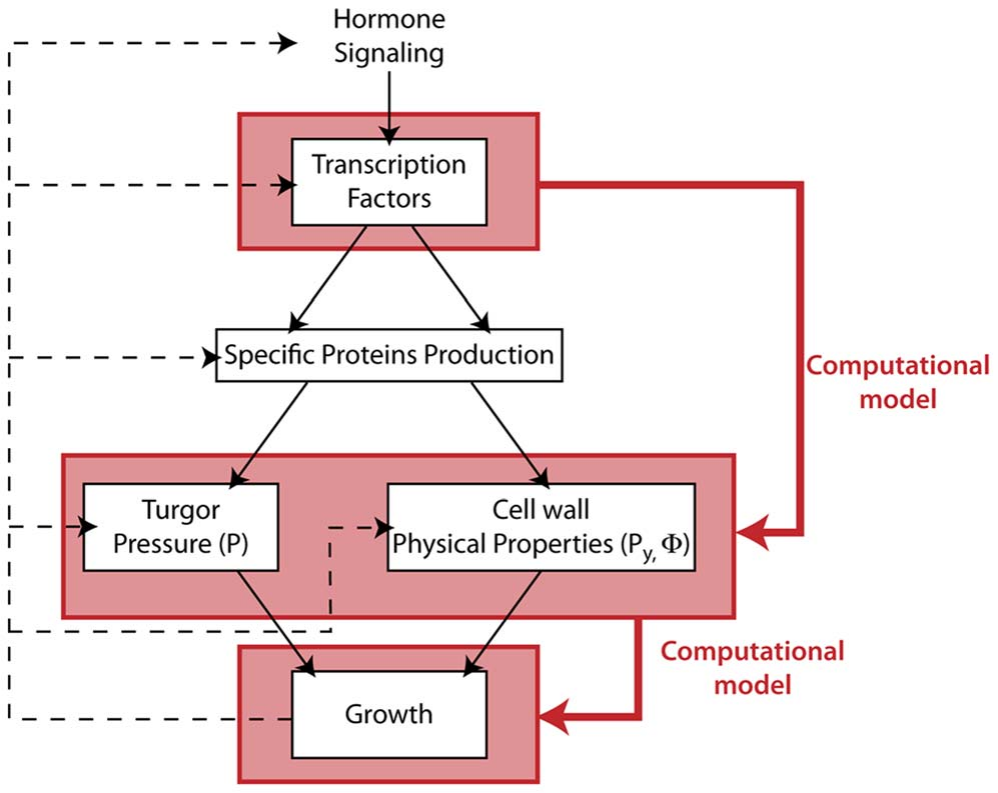
Schematic view of the regulation of growth in multicellular tissues. The different horizontal layers represent different levels of biological organization. The plain black arrows symbolize the downward stream of regulation between growth hormones and actual growth through transcription factors activation and physical quantities modulation. The red plain arrows depict the indirect, integrated relationships between transcription factor activation, physical quantities modulation and cell wall irreversible extension our computational framework attempts to grasp. Finally the black dashed upward arrows stand for possible feedback mechanisms from shape changes on the biochemical regulation of growth.

In a multicellular context, morphogenesis relies on differential growth across tissues. Each cell may feature specific values for the various parameters (turgor pressure, yielding threshold, extensibility…) used in Eq.(1). In principle, the regulation and coordination of these parameters is achieved through the action of the molecular regulatory networks that control the composition and mechanical properties of the cell wall, as described by the black arrows in Fig. 1. For example, cell wall modifying enzymes such as expansins, xyloglucan endo-tranglycosylases or pectin modifying enzymes are known to be triggered by transcription factors such as APETALA2 [9], MONOPTEROS [10] and AGAMOUS [11]. At the scale of the cell wall, actions of such enzymes have the potential to increase or decrease the viscosity and/or the rigidity of the wall. As a consequence, extensibility 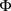 in Eq.(1) may be modified and affect growth.

Although the general concepts described above are widely accepted, they do not explain how genetic determinants collectively generate an organism with a particular shape. The situation is made even more complex because morphogenetic events at the multicellular level can feed back on the cellular or molecular scale. Morphogen gradients, for example, are limited by the geometry of the tissue in which they diffuse [1], [2] and mechanical stresses generated by differences in growth rate within an organ can potentially feed back on cellular growth directions and rate [3]. It is therefore not self-evident to explain how a particular gene by interfering with local cell wall properties influences the overall shape of an organ. To proceed further and to explore hypotheses linking in an intricate way gene function to morphogenesis, a computational modeling framework is required (red bold arrows in Fig. 1).

In a seminal series of papers, Coen and colleagues have proposed such a framework for tissue growth, termed the Growing Polarized Tissue (GPT) framework, that can capture overall growth rates and directions of tissues in three dimensions while taking into account mechanical interactions between different regions ([12]–[14]). With this method, the authors were able to propose hypotheses for the genetic regulation of organ formation in different species [14]. This framework was the first system able to simulate 3D organ development and shapes based on plausible genetic regulation hypotheses. However, in GPT gene functions are expressed in relatively abstract terms. This is mainly because it models events occurring at the scale of entire tissue regions. Recently, several attempts were made to develop other modeling frameworks at cellular resolution and to build mechanical models of morphogenesis for multicellular tissues. To reduce complexity, these models have been initially restricted to 2 dimensions either in the plane ([15] for algae, [16], [17] for leaves and [16], [18] for roots), or on 2D surfaces in 3D ([3] for shoot apical meristems). However, new technologies to image and segment the complete volume of multicellular tissues in 3D at wall resolution [19] and to measure cell mechanics [20], [21] now lead to new questions regarding the interactions between cells in three dimensions. Addressing these questions, requires a new generation of models, able to account for the genetic regulation of biophysical processes in 3D multicellular systems. Here, we present such a modeling framework where cell growth results from the deformation of walls that are under tension in the tissue. A tensorial formalism is used to account for the anisotropic nature of cell wall material and to model wall deformation based on a generalized Lockhart viscoplastic law. An adapted finite element method has been designed to carry out efficient numerical simulations. This computational framework is then used to analyze the development of the early flower bud and test the effect of different regulation hypotheses.

## Results

Prior to describing our computational framework to model multicellular tissue growth, let us analyze the physical situation of a small region - a cell or a portion of a cell wall - in a growing tissue.

### Multicellular growth involves both cell autonomous and non-cell autonomous forces

**Figure 2:**
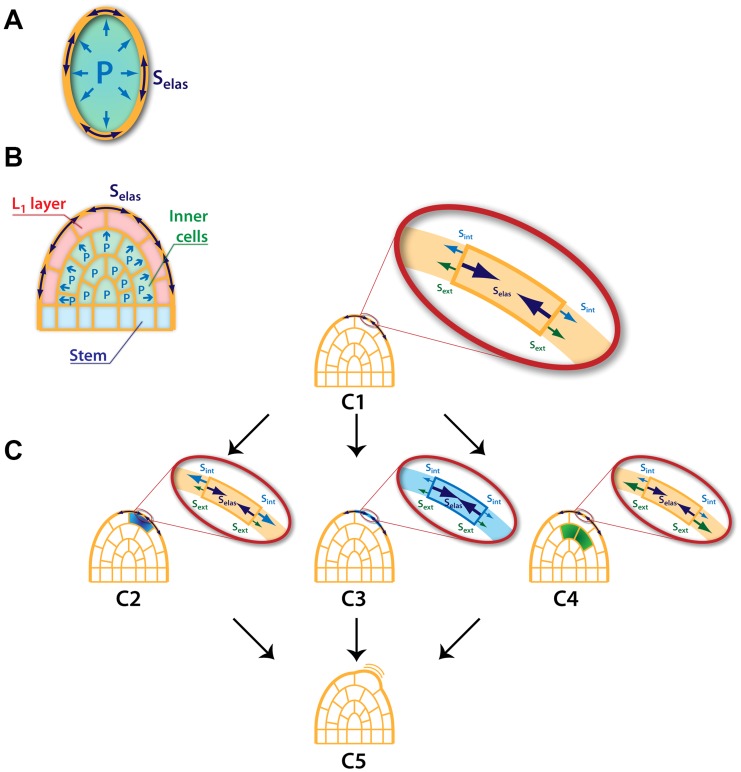
Origin of forces driving growth in a multicellular tissue. (A) In the single-cell case, the mechanical (elastic) stresses (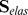, dark blue double arrows) undergone by the cell wall are due to the inner pressure (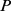, light blue single arrows) of the cell. The mechanical equilibrium within this wall is regulated by the cell itself. (B) In a tissular case, (here a shoot apical meristem), mechanical stresses 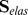 within the outer cell walls of the L1 layer (light red cells), can be modulated by remote cells (here in light green). In this case the stem (light blue cells) plays the role of a base on which the inner cells rely in order to push the L1 layer upward. (C) Three main modalities of growth can be considered in a multicellular context (details on the stresses equilibrium within the outer cell wall are represented in the zooming views). From an initial state (C1) of the growing tissue three scenarios are considered: (C2) & (C3) present cell-autonomous ways where growth of a given cell is triggered by an increase of its inner pressure or a modulation of its wall mechanical properties respectively. (C4) represents a non-cell-autonomous case in which growth of the studied cell is initiated by physical alteration of its neighbors. (C5) All three modifications result in the local outgrowth of the considered region.

Similarly to isolated cells, cells in plant tissues are growing under the action of forces that stretch their wall and make it yield. However, for single isolated cells, the only significant forces able to trigger growth are the ones generated by its turgid cytoplasm pushing on the cell wall (Fig. 2A). In a multicellular context, the situation is somehow complicated by the fact that cells are rigidly connected to each other. The deformation of one cell generates physical constraints on its neighbors and vice versa. It has been recognized by Sachs as early as in 1882 (cited by Kutschera [4]) that epidermis cells in plant tissues are experiencing external forces due to the inner turgid cell layers pushing outwards against the surface cells, and inducing tension stresses in the epidermis (Fig. 2B). In a plant tissue, the mechanical stresses undergone by one cell wall are thus not only due to their own turgid cytoplasm, that we call *cell autonomous stresses*, but also comprise the physical constraints imposed by the neighboring cells, called the *non-cell autonomous stresses*.

Genes can regulate growth by controlling processes that modulate these stresses. Cell autonomous stresses can be modulated directly by the cell itself. This can be done in two ways: either by increasing the cell's own turgor pressure or by modulating the mechanical properties of its wall (e.g. by changing its elasticity or its yielding threshold). In contrast, non-cell autonomous stresses reflect distant mechanical interaction between cells where genes are being expressed and the ones where the consequences of this expression are observed.

Let us illustrate these different possibilities in the context of a primordium outgrowth on a growing meristematic dome, see Fig. 2C. As discussed above, any specific cell of the epidermis is suject to three types of stresses: *i*) the stress 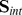 induced by the cell's inner turgor pressure on the wall *ii*) the stress 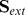 resulting from the action of the rest of the tissue on the cell wall and *iii*) the stress 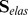 due to the cell wall elastic deformation in reaction to the other stresses. In growing tissue, mechanical equilibrium leads to a balance between these three stress components (Fig. 2C1):
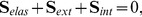
(2)


In a cell-autonomous perspective, regulation of the inner pressure of the cell would lead to a change in 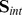 (Fig. 2.C2) and modifying the mechanical properties of the wall would directly impact the elastic term 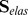 (Fig. 2.C3). Likewise, modulating 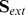 (e.g. by changing the turgor pressure of some neighboring cells) would correspond to a non-cell autonomous regulation of growth, (Fig. 2.4). Interestingly, new experimental evidence of the possibility of such a non-cell autonomous regulation has recently been reported by Peaucelle *et al.* [20].

The situation is complex as, if one of these stress components is affected by some regulation mechanism, all the other stresses will in turn be affected. Moreover, in real plants several of these regulations may be triggered at the same time, leading to even more complex interactions between regulation and growth. Therefore, to understand the growth of multicellular tissues, one needs to model both cell autonomous and non-cell autonomous growth. How can this be achieved?

### Computational framework to model local deformations in tissues

**Figure 3:**
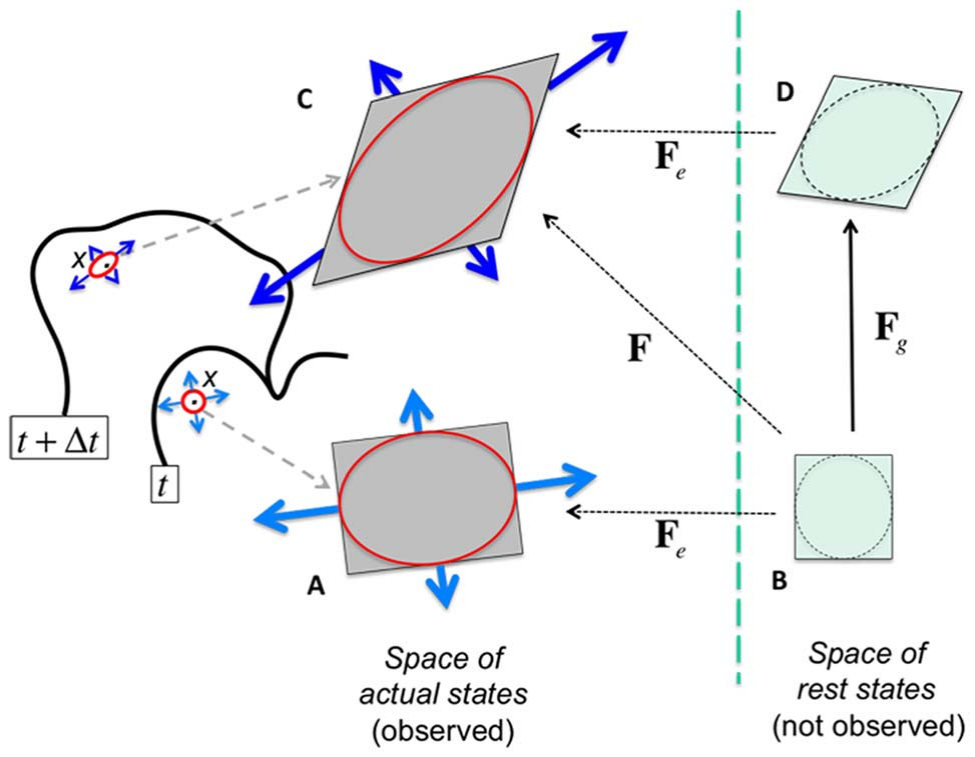
Formalization of plastic growth of a small region of wall. A tissue region is in general observed as a deformed object in a real tissue (A) due to local stresses internal to the tissue (light blue arrows). Taken outside its tissue context, without any stress on its borders, the region has a rest shape (B). Note that this rest shape is not actually observed. The transformation matrix to pass from the rest shape to the observed deformed shape is denoted 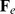. Due to changes in stress distribution in time, at a subsequent date the stress configuration acting on the region changes (dark blue arrows) and induces a new deformation of the region (C). If the intensity of the elastic deformation between the former rest shape (B) and the new deformed object (C) is above a certain threshold, then plastic growth is triggered: the rest shape is remodeled by the cell by adding material to the wall (D) which reduces the elastic strain. This change is made according to a constitutive rule that describes the material plasticity (see Model section below). As a result, the transformation 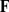 from the old rest state (B) to the new deformed state has been decomposed as a product of a reversible term 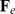 and an irreversible term 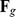 representing growth.

Both cell and non-cell autonomous growth rely on turgor-generated forces that are directly translated into mechanical stresses within the cell walls. Therefore considering a mechanical stress-based growth mechanism ensures that both types of growth are taken into consideration. Depending on their mechanical properties, the cell wall deform in response to the direction and intensity of these stresses. We assume that each small wall region of each cell in the tissue, at any time 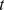, has a rest shape, i.e. the shape that the region would have if isolated from the rest of the tissue. Under the effect of the tissue stresses due to turgor pressure and connection to other cells, each small region is elastically deformed with respect to its rest shape. For the sake of simplicity, we assume that the thickness of the wall is kept at a constant value during growth (we do not model the details of the wall remodeling process itself). We also assume that the thickness of the walls has no major mechanical effect at this scale of analysis and therefore can be integrated in the wall's in-plane properties. Then, if the region is chosen sufficiently small, the wall deformation can be assimilated to an affine transformation and represented by a matrix 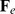 called the deformation gradient (Fig. 3*A*-*B*, see Model section for mathematical details). From 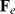, it is easy to compute the region strain 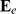:
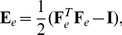
(3)


where 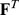 denotes the transpose matrix of 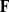 and 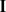 the identity matrix. The fact that this strain is the elastic response of the material to tissue stresses is described by a constitutive law of the wall material. In the simplest case, it is a linear relation between elastic strain and stress corresponding to a generalized Hooke's law in 3 dimensions:
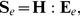
(4)


where 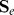 is a matrix representing the stress on the small region and 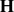 is an order-4 tensor expressing the local elastic properties of the material. In particular, the anisotropy of the material if any is encoded in 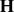 coefficients.

The forces that act on a region may vary throughout time, notably through either direct or indirect genetic regulation. Regions may be subject to new stress distributions (Fig. 3*C*), inducing new strains. In the spirit of Lockhart equation for cell volumes (Eq. 1, we assume that if the strains get above some threshold, the walls start to yield and the cell to remodel them. As a consequence, the reference state of the region is modified irreversibly (Fig. 3*D*). A number of studies have proposed to model this process by using a multiplicative decomposition of the overall deformation 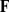 [22] and then [23], [24] (Fig. 3*B*-*C*-*D*),
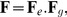
(5)


where 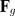 denotes the irreversible modification of the rest shape of the region, called the growth tensor, and 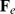 is by a purely elastic deformation corresponding to the reversible part of the process.

### The equation of growth

To describe growth we need a constitutive law that relates the rate of change of the matrix 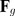, called growth rate tensor, to physical processes. We assume this law to be strain-driven: above a certain deformation threshold, the rest configuration of a region changes at a speed proportional to the strain of the region. In terms of tensors, the simplest form of such a law can be expressed as (see Model section for details):
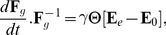
(6)


where the left-hand term defines the relative rate of variation of the reference state, 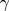 is a constant characterizing the rate at which walls yield (extensibility) and the components of the term 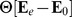 correspond to non null tensor components in the directions where the elastic strain is above the threshold values encoded in 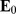. Replacing the strain components in Eq.6 by their stress counterpart using Hooke's law, leads us to the following generalized 3D *Lockhart-like* form:
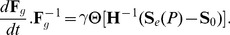
(7)


This equation shows how growth is related to key mechanical variables: the extensibility 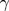 controlling the rate of growth, the elastic properties of the material 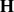, the turgor pressure 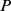 that appears through the stress it induces 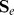 in the tissue and 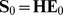, a plastic stress yielding threshold corresponding to the strain yielding threshold 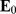. Note that the mechanical properties of the material are taken into consideration through a rigidity tensor 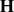 which allows the plastic deformation evolution (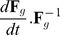) to be non-collinear to the plastic stress (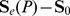).

The above equation describes the plastic deformation evolution of a small region of the tissue, typically a part of a wall, during a small amount of time. To compute the deformation of the whole tissue during development, we need to integrate these local deformations over the whole tissue and throughout time, so that all these elements are assembled in a symplastic manner in the deformed tissue. This computation is made by minimizing the global mechanical energy in the tissue (see Model section). The strain and stress configuration of each region will thus be chosen so that the mechanical energy is minimal among all possible combinations of local elastic deformations that preserve the integrity of the tissue and the adjacency of cell walls. By contrast, the rest configuration of each individual small region is not necessarily compatible with that of other regions [25], i.e. there may not be physical continuity between rest configurations. The integration is carried out using a finite element method - FEM - (see Model section below).

### A comparative analysis of the putative mechanisms behind organogenesis at the shoot apical meristem

**Figure 4:**
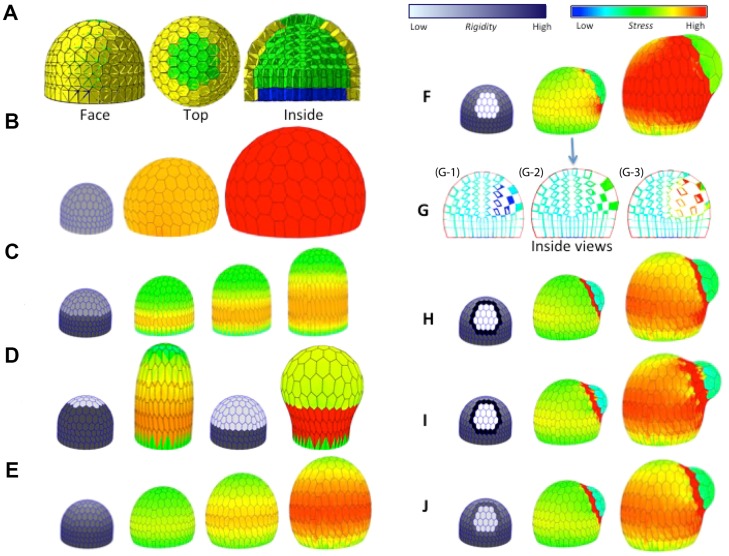
Growth regulation mechanisms and their impact on shape development. (**A**) Face, top and inside view of an artificial dome made of cells with mechanical properties. The transversal cut shows the inner cells. The basal faces of cells shown in blue here are constrained to keep in a horizontal plane. (**B-E**) Growth of a multi-cellular dome. In all the simulations, the gray scale code on the initial dome represents regions with different rigidities. A different color code is then used on the other steps to figure mechanical stress intensity, *c.f.* color scale on the top right corner. (**B**) Homogeneous dome: all cells are isotropic with identical elasticity, plasticity threshold and growth speed. (**C**) Mechanical anisotropy is imposed on the lower half of epidermis to model the effect of microtubules circumferential orientation. Axial growth emerges. (**D**) Analysis of the extent of the anisotropic zone on growth. From left to right: Initial state of the simulation with circumferential anisotropy imposed up to 80% of the dome height: The resulting growth is axial. Initial state with a dome anisotropy limited to 40% of the dome height: The corresponding growth is globular. (**E**) Growth with a gradient of circumferential anisotropy from the bottom to the top of the dome: The resulting growth is inbetween purely axial and isotropic. (**F-J**) Creation of a lateral dome. (**F**) The rigidity of the cells in a small region at the flank of the meristem is decreased (cell autonomous regulation). During growth a lateral bump starts to form. The simulated dome is shown at two time points (middle and right). (**G**) Transversal cuts of a dome showing tentative generations of a bump with non-cell autonomous stresses: (G-1) Decreasing wall rigidity (10-fold) in a group of inner cells (blue cells with *i.e.* low mechanical stress): No visible bump emerges; (G-3) Increasing the turgor pressure (3-fold) in the same group of cells (red cells *i.e.* high mechanical stress): A shallow bump emerges and inner tissues are compressed inside. Compare with the reference situation (G-2) corresponding to a transversal cut of F middle. (**H**) Similar to F, but cells surrounding the primordium region are made stiffer. A well marked dome appears (middle and right). (**I**) Similar to F, but cells surrounding the primordium region are made stiffer in the bump ortho-radial direction only (anisotropy in boundary region). (**J**) Simulation similar to H, combining a smaller decrease of rigidity with an increase of the walls synthesis rate (namely extensibility) in the primordium. Movies corresponding to each simulation are available as Supporting Information.

We next used our modeling framework to analyze organogenesis at the shoot apical meristem (SAM). The SAM is a population of stem cells that continuously initiates new stem tissues and lateral organs, thus generating all the aerial parts of the plant. We first constructed a model of the SAM as a dome made up of polyhedra representing the 3-D cells and rigidly connected to each other (Fig. 4*A*). The faces of these polyhedra represent cell walls and are composed of 2-D elastic triangular elements whose mechanical properties are represented by tensors 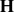. The stiffness of these elastic triangles is set higher in the epidermis walls than in the inner walls and may be either isotropic or anisotropic for epidermis triangles. We assume that cells are inflated with a uniform turgor pressure 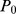, and that triangle mechanical properties are all initially isotropic and uniform.

In this initial configuration, the turgor pressure induces a stress that puts all the cell walls under tension. If the plastic growth threshold 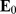 is reached, the dome grows isotropically in all the directions (Fig. 4*B*). This plastic deformation is accompanied by a decrease of inner pressure in cells as their volume increases due to growth. *In vivo*, this would lead to a difference in water potential between the cells within the dome and surrounding tissues, drawing new water from below the dome inside the meristematic cells. It is generally assumed that water uptake is combined with osmolyte regulation to maintain turgor relatively constant in the cell during growth [4]. As these processes are considered fast compared with growth, we simply assume in our model that, otherwise stated, the turgor pressure is continuously kept constant at 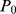.

A key feature of meristematic activity is the generation of cylindrical stems and roots. In principle there are several ways to generate axial structures, but there is overwhelming evidence that this is due to a switch from isotropic to anisotropic growth over several cell files. Indeed, when cells leave the meristematic dome, they start to generate cellulose microfibrils in highly ordered arrays, oriented along the circumference of the meristem [3], [26]. This microfibril organization creates a high circumferential rigidity that favors growth in the perpendicular axial direction. This mechanical anisotropy can be reproduced in the 3D model by imposing higher rigidity in circumferential direction using anisotropic 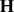 tensors (Fig. 4*C*). Interestingly, the ability of the microfibrils to create well formed axes depends on the amount of microtubule anisotropy in the dome (Fig. 4*D* dark gray). If too small, a bulge forms at the tip (Fig. 4*D*). Intermediate shapes can be obtained by using a less sharp transition between isotropic and anisotropic regions, using e.g. a gradient of anisotropy between the bottom and the top of the dome (Fig. 4*E*).

Next, using this mechanical model of the SAM, we tested different scenarios for organ emergence. As discussed above, several processes can in principle account for this phenomenon, in a cell- or non-cell-autonomous way, including changes in turgor pressure, modifications in wall stiffness and modifications in the rate of wall synthesis.

Starting from the growing dome of Fig. 4*E*, we first tested the possibility to grow an organ on the dome assuming cell autonomous regulation. For this, we lowered the outer periclinal wall rigidity in a small region close to the tip of the main dome (Fig. 4*F*). This created a bulging zone in the corresponding region of the tissue. We then tested the alternative possibility to create a primordium in a non-cell autonomous manner, by relaxing the wall rigidity up to 10-fold in a group of cells immediately below the previous surface region. No visible bulges could be obtained in this way (Fig. 4*G*–1; compare with Fig. 4*G*–2 corresponding to a transversal cut obtained from the simulation of Fig. 4*F* middle that shows a much larger bump at the same stage of development). To further explore the ability of non-cell autonomous stresses to trigger bump outgrowth locally, we also tested the possibility to obtain a bulge by increasing (by up to a 3-fold factor) the turgor pressure in the same group of inner cells. Here again, no clear bulge could be obtained (Fig. 4*G*–3). In this latter case, we could observe in the transversal cut that the tissues were actually compressed internally. We concluded from this series of simulations that bumps can more easily be generated in the context of cell autonomous regulation.

In this context however, we could observe that the growing bumps were not clearly separated from the main dome (e.g. Fig. 4*F*). We therefore constrained a ring of cells at the surface, around the bump location, to be very rigid. This resulted in a well formed bump growing on the top of the initial dome (Fig. 4*H*). A similar separation between the two organs can be obtained by stiffening the cells of the ring only in the circumferential direction. Interestingly, the cells of the ring are also supporting high stress but are left free to grow in the axial direction of the new bump (compare right images in H and I). A well marked bump can also be created by a less important decrease of the primordium zone rigidity compensated by a local increase of the cell growth rate in this region (Fig. 4*J*).

The simulations showed how simple scenarios can in principle explain important morphogenetic processes during plant development, including the formation of cylindrical stems and roots and the outgrowth of lateral organs at the SAM. Importantly, although these simulations can lead to comparable shapes, they make different predictions regarding the mechanical properties and resulting growth patterns.

### A case study: Morphogenesis of a flower bud

**Figure 5:**
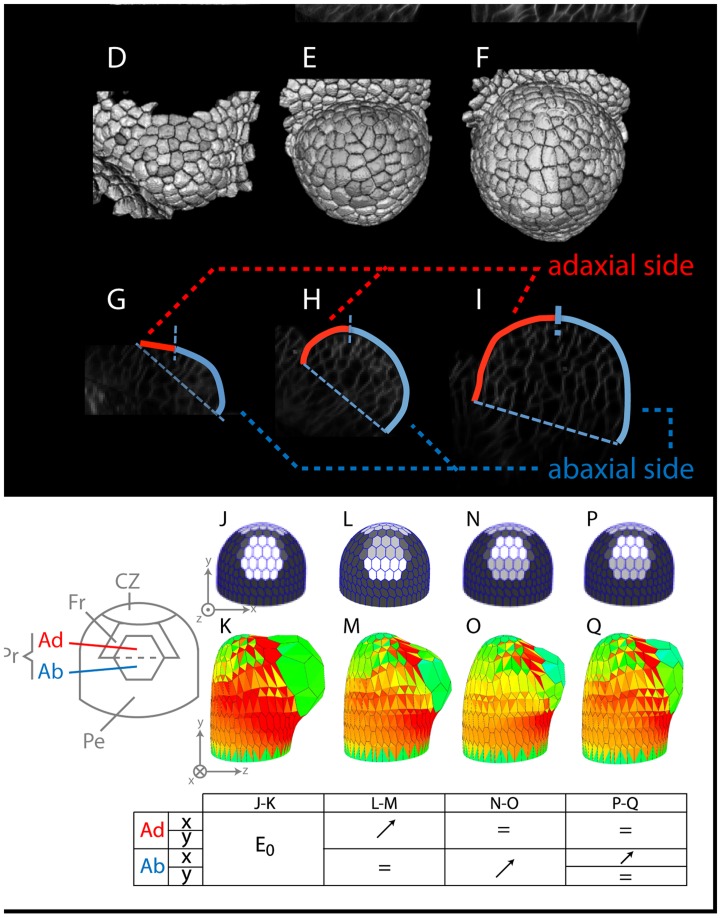
First stages of development of a flower bud. **Upper part**: (**A-B-C**) Transversal sections in the young outgrowing flower bud at time points separated by 24 h. (**D-E-F**) Automatic 3D segmentation of the corresponding confocal images using the MARS-ALT pipeline [19]. (**G-H-I**) The analysis of growth patterns shows that growth at the abaxial side is faster than at the adaxial side, causing the floral meristem to bend towards the SAM. **Lower part**: Different attempts were made to regulate the mechanical parameters in time so as to reproduce this differential growth behavior. On the left:representation of the zones used in the simulation (CZ  =  Central Zone, Fr  =  Frontier, Pr  =  Primordium, Ad  =  Adaxial zone, Ab  =  Abaxial zone, Pe  =  Periphery). For all the simulations, the rigidity was decreased (light gray) in Pr (relative to CZ and Pe, and in the anisotropic zone Fr, the direction of maximum rigidity was set ortho-radially to Pr. With such an initial configuration, a globular and symmetric dome emerges normal to the surface (**J-K**). Then by tuning the mechanical properties of the Ad/Ab regions we could obtain different asymmetric developments: increasing the rigidity of Ad cells (medium gray) resulted in a restricted development of the upper part of the primordium (**L-M**) while, by contrast, an increased rigidity of the Ab cells (medium gray) shifted the primordium development upwards (**N-O**) as expected. Finally a growing dome with correct development of the Ad/Ab regions could be obtained when the abaxial cells where also imposed a high degree of anisotropy (orientation shown by the thick black bars oriented circumferentially in the Ab, (**P-Q**)). The table under the snapshots illustrates the relative variations of Elastic modulus used for each case. The x and y coordinates respectively refer to the axial and circumferential directions, as exposed on sub-figures (J) and (K). Numerical values used in the simulations and corresponding movies are available as Supporting Information.

The simulations presented above were based on abstract versions of real meristems. We therefore next applied our computational framework to perform simulations from realistic templates. Hereby, we used the floral meristem of *Arabidopsis thaliana* - which has been very well characterized - as a case study. As a reference for model construction, we used a series of confocal stacks of the same young growing floral primordium taken at 24 h intervals from early stage 1 to stage 2 (3 time-points, Fig. 5*A*-*B*-*C*). Using the Mars-Alt pipeline [19], the individual cells were identified and cell lineages were tracked in the thus segmented reconstructions (Fig. 5*D*-*E*-*F*). The confocal images and 4D reconstructions suggest that *in vivo*, the primordium first grows out from the meristem as a small radial symmetric globular structure in a direction normal to the surface of the meristem. Then the global direction of primordium growth changes progressively and the initial symmetry around the normal to the surface breaks as the the abaxial region expands more rapidly than the adaxial region (Fig. 5*G*-*H*-*I*). The sepals appear with different growth rates as the abaxial and adaxial sepals grow much faster than the two lateral ones. A recent analysis of gene expression in the flower bud showed complex spatio-temporal expression patterns with as many as 16 different domains expressing different combinations of transcription factors [27]. This raises two main questions: (i) how many different gene activities would in theory be required to produce this structure? (ii) are mechanical-related actions of these genes alone sufficient to reflect for the observed shape evolution?

To address these questions we investigated with our mechanical model how to reproduce, at first qualitatively, the developmental pattern described above. Hereby we distinguished two phases: (a) the initial outgrowth of the bud and (b) the formation of the sepal primordia. For this purpose, we made a number of assumptions based on the literature and on the first set of models described above.

First, we simulated the outgrowth of the flower bud using the following itinerary through successive scenarios:

**i** In the first scenario, we tested the possibility to initiate organ outgrowth by a combination of local wall softening in the incipient primordium and anisotropic wall stiffening in the organ boundaries. A number of experimental evidence supports such a scenario. The young primordium is characterized by relatively high concentrations of the plant hormone auxin [28], [29]. There is evidence, that auxin influences cell wall loosening by the activation of specific enzymes. For instance, in the hypocotyl ABP1 (Auxin Binding Protein 1) controls the level of expression of glycosyl hydrolases known to modify hemicellulose within the cell wall, [30]. At the apex, atomic force microscopy measurements suggested that auxin accumulation leads to cell wall softening via a PMEI3-dependent pathway [31]. Finally, transcription factors such as AINTEGUMENTA and MONOPTEROS which are homogeneously expressed in the young outgrowing flower bud are thought to regulate the expression of expansins and xyloglucan modifying enzymes [9], [10]. Organ boundaries are also characterized by specific gene expression patterns. In particular the CUP-SHAPED COTYLEDON transcription factors are strongly expressed between the meristem and the primordium and genetic studies show that they repress growth in this region (e.g. [32], [33]). In addition, Hamant et al. [3] found that in the same region, cells are likely to have highly anisotropic wall structure. Based on these observations and on preliminary simulations shown on Fig. 4*I*, we assumed the existence of a band of anisotropic cells around the primordium's upper half. As a result, the model produced a bulge normal to the surface with a quasi symmetric shape (Fig. 5*J*-*K*).

**ii** The next morphologically significant step in organogenesis happens when differential growth behavior between the adaxial and the abaxial regions emerges. At stage 2 (or P2) cells of the abaxial side of the primordium start to expand faster in the meridional direction than the ones from the adaxial region, (Fig. 5*G*-*I*, see also [34]). To account for that differential growth in our simulations, we assumed that mechanical properties are differentially regulated between the abaxial/adaxial regions. Although there is no strict evidence for differences in mechanical properties, both regions are characterized by specific gene expression patterns early on. For instance, transcription factors such as FIL, KANADI, YABBI or ARF3 and ARF4 are part of interlocking pathways necessary for organ polarity and proper development [35], [36], for reviews see also e.g. [27] and references therein. To account for the faster growth rate in the abaxial region, we first assumed that the abaxially expressed genes caused a lower rigidity in that region (Fig. 5*L*-*M*). This indeed resulted in a developmental abaxial-adaxial asymmetry. However, the organ tended to develop downwards, opposite to what is observed *in vivo*.

**iii** To correct this behavior, we set rigidities in the primordium zone in the opposite way, i.e. with higher rigidity now in the abaxial region. The summit/adaxial region was now developing faster toward the upper direction but, contrary to observations in actual development (Fig. 5*G*-*I*), with little shape asymmetry (Fig. 5*N*-*O*). Indeed, *in vivo* observations carried out by e.g. Long et al. [34] and Fernandez et al. [19] were reporting anisotropic growth in the abaxial side of the floral primordium. The discrepancies in simulations of Fig. 5*N*-*O* could finally be corrected by setting a high rigidity anisotropy in the abaxial cells. (Fig. 5*P*) The dome asymmetry was then correctly oriented upward (Fig. 5*Q*), with fast development of the abaxial region upwards as expected.

**Figure 6:**
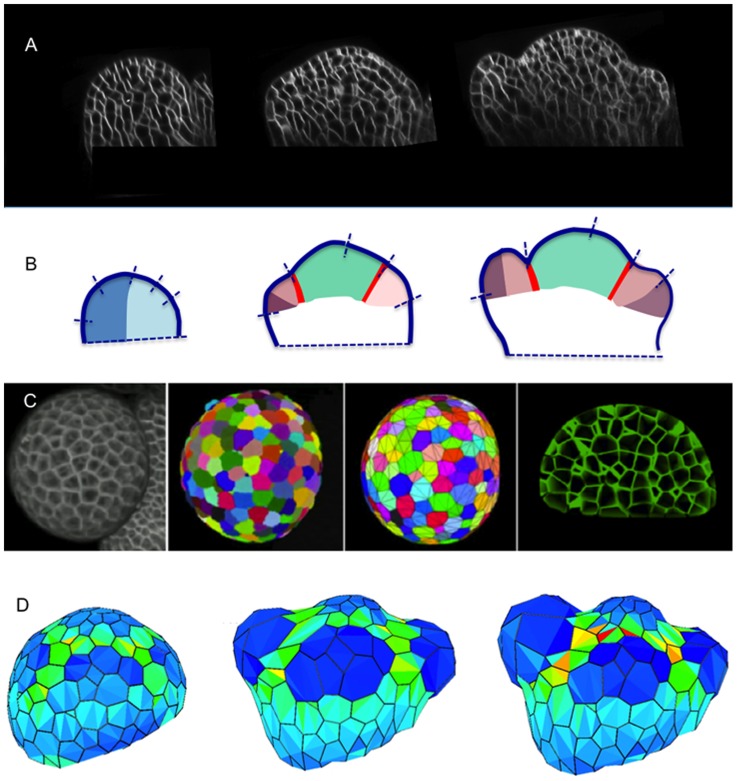
(A) Transverse sections of confocal images showing floral bud development between stage 1 and early stage 3. Abaxial sepals start to grow out first (middle and right image). (B) Growth patterns and gene expression profiles. The respective development of the different zone is indicated by small bars at the meristem surface. This growth pattern is accompanied by a change in gene expression patterns. At stage one, the floral bud is characterized by adaxially (light blue) and abaxially (dark blue) expressed genes. Other genes such as LFY and ANT are first expressed throughout the young flower. When the sepals start to grow out abaxial and adaxial domains are again established in these young organs (resp. dark and light pink), characterized by specific expression patterns (e.g. REV or FIL). Other genes, such as ANT or AHP6 will finally remain active throughout the pink zones that will generate the sepals (dark and light pink). Boundary zones, characterized by genes like CUC (red) separate the primordia from the meristem proper, where genes like STM (green) are active. For review of expression patterns see [27]. (C) Creation of a 3D geometric model of a flower bud. From left to right: confocal image; automatic cell segmentation using Mars-Alt pipeline [19]; construction of a mesh based on cell vertices; transverse section of the mesh showing the geometric representation of the inner layers. (D) Mechanical simulation of a flower bud development and its regulation by genes. Progression in the flower bud development is shown at three different stages, from primordia initiation to early stage 3 (see Supporting Movie S5).

Following on the simulated flower bud growth, we next simulated the outgrowth of sepal primordia (Fig. 6*A*-*B*). To initialize the FEM simulations we constructed a triangle-mesh of the floral meristem at stage 2 based on the segmented image. The vertices of triangles coincide with the cell vertices (Fig. 6*C*). We then reused the mechanical setting of this model to grow the four sepal primordia on the outgrowing dome obtained in phase (a). *In vivo*, this stage is characterized by auxin accumulation at the four sites of sepal initiation [37], followed by the activation of genes that promote organ outgrowth - in particular AINTEGUMENTA - first in the abaxial and adaxial sepals, then in the lateral ones (Fig. 6*B*). Simultaneously, organ boundary genes are activated at the flower meristem boundary and in between the primordia. As for the flower bud simulations presented above, we translated these gene expression domains into zones with different mechanical properties, i.e. wall loosening in the primordia and a anisotropic stiffening in the boundaries as described above. Again the rigidity in the abaxial regions was set higher than in the adaxial ones. We also assumed that the initiation of lateral sepals was slightly delayed with respect to the adaxial/abaxial pair. Based on initial geometric structures obtained from confocal microscopy, the resulting simulation was able to reproduce the developmental dynamics of a flower bud and its first lateral organs based on a spatio-temporal synchronization of the changes in mechanical properties of regions (Fig. 6*D*).

## Discussion

Modeling strategies are increasingly used to help understanding the mechanisms behind shape changes and to make predictions. However, so far models have been either relatively abstract, by focusing on the instructing role of the gene network rather than on the physical constraints of cell growth, or well-defined biophysically but weakly connected to the gene network and its regulatory role. In addition, models able to simulate in a realistic way in 3 dimensions the development of organs on the basis of cellular regulation were missing.

Here we present such a conceptual and modeling framework that is able to integrate the genetic input and the mechanical determinants of the cell in a 3D multicellular context. Using this framework, we tested different scenarios relevant to shape changes and made a number of predictions for organogenesis at the shoot apical meristem. Importantly, our approach does not overlook the complexity that relates to multicellularity, but fully incorporates it by taking into account both cell autonomous and non-cell autonomous forces in 3D tissues.

In the past decade several modeling frameworks have been proposed that are aimed at providing insight at multiple levels of organization and regulation [8], [13], [16], [18]. The framework we propose here presents three significant complements to these existing models. First, we explicitly formalize growth of plant cells in tissues taking into account our current knowledge on the mechanisms controlling growth at the cellular level. The resulting equation allows us to make precise prediction on the primary function of molecular regulators in terms of four local parameters i.e. turgor pressure, wall extensibility, wall elasticity, and yield threshold. Second, taking advantage of both the availability of increased computer power and novel computing techniques we were able to design a model tissue with cellular resolution in 3D. This integrates a new data structure to handle multicellular tissues [38] used by other groups for tissue simulation (e.g. [18]) and the use of the modeling software platform SOFA for mechanical simulation in biology [39]. The platform makes it possible to express the mechanical, geometrical and simulation components in a completely modular way and optimizes the computational overhead during simulations by making use of efficient implicit integration solvers. As a result, simulation in 3D of flower development can be achieved in near interactive time. The model is able to handle several hundreds of cells, and can take into account both biochemical and physical properties of cells. Third, by fully accounting for the complexity behind multicellularity, this framework allows us to compare the contribution of cell autonomous forces and non cell autonomous forces in shape changes. Our conceptual framework notably integrates the mechanical outputs from the gene regulatory network in each individual cells and the secondary effects deriving from growth of neighboring cells. It also provides us new means to quantitatively investigate the contribution of geometrical, biochemical and mechanical interactions in growth regulation with cell wall resolution.

The simulations carried out on both synthetic and realistic templates pose a number of interesting questions that now have to be addressed experimentally. First, the plant seems to have the possibility to create similar shapes through different pathways. Growth rates in the organ boundaries, for example, can be restricted by modifying wall stiffness, wall anisotropy or combinations thereof. In parallel, organ outgrowth can be promoted by reducing wall stiffness and/or by increasing the rate of wall synthesis. Interestingly, the model may help us establish a hierarchy between developmental scenarios and organize the plausibility of developmental scenarios and to organize accordingly experimental investigations. For example, to create a realistic primordium on the meristem dome, simulations clearly indicated that the intuitive solution consisting of softening the abaxial zone is the least plausible (Fig. 5*L*-*M*). This forced us to explore other, less intuitive hypotheses consisting instead of loosening the adaxial zone (Fig. 5*N*-*Q*), and that appeared to be more realistic. Another interesting outcome concerns the number of different gene activities that are required to generate an organ primordium or a flower bud. Whereas gene expression studies have revealed a complex partitioning of the growing flower bud in different domains, the simulations carried out on realistic templates, suggest that only five domains (central zone, peripheral whorl, abaxial and adaxial domains and boundary) with specific wall modifying activities would be sufficient. These hypotheses should now be tested. This will include classical approaches such as gene expression studies and transgenic approach, but also more challenging techniques required for quantitative growth analysis. At this stage, the simulations already suggest a number of relatively straightforward experiments. Differences in wall stiffness between adaxial and abaxial domains can be measured, for example using atomic force microscopy [21]. Correlations between organ outgrowth and the expression of genes involved in wall modifications can also be made. Modifications in wall anisotropy at the abaxial side of the primordia -as suggested by the model - can even be monitored in vivo by direct observation of microtubule dynamics [3]. Metrics will also have to be developed to compare quantitatively results of mechanical simulations and the observed, actual geometry of developing organs. Based on the recent progresses in imaging protocols, cell segmentation and tracking softwares, e.g. [19], [40], it will now become possible to routinely compare the simulated development of particular cellular regions (e.g. central zone, primordium, abaxial/adaxial, frontier zone,…) with the observed ones based on a quantitative comparison of their principal direction of growth, rate of growth, and of various shape factors such as local curvature, degree of symmetry, compacity, *etc*. In turn, this opens the way to the development of inverse modeling techniques, where the estimation of some mechanical parameters that are difficult to measure, will be derived from minimizing the distance between simulated and observed developing shapes.

The current version of the model also has some limitations. In particular, it currently does not include cell division. Generic rules for cell division have been proposed in the literature, e.g. see recent works [41], [42] and references therein. However, in the meristem, the validity of these rules has not been thoroughly assessed, and in particular, the way cells divide in the inner layers of meristem is still mostly unknown. Imposing specific rules here would add an extra level of assumptions whose effect on shape development would require specific analysis. In addition, the design of a robust algorithm for cell division on real 3D meshed tissues is a challenging issue in itself and would require the development of robust 3D algorithms. For these reasons, we restricted in this paper our analysis to time-lapses that are small enough to consider that deformations and mechanical forces are not markedly modified by cell division. We used simulation duration such that the deformations of cells were kept reasonably small (cell volumes increased by less than a 3-fold factor). A second limit of the current model is related to the use of 2D finite elements with no thickness for the representation wall parts. We considered that at least in a first approach neglecting the effect of the third dimension (thickness) of these elements and the associated flexural stiffness was a reasonable approximation. A further step would then consist of using more complex 3D shell elements in the numerical method to account for situations where these non in-plane stresses cannot be neglected. Finally, in further rounds of modeling, additional hypotheses and levels of regulation can be included as well. For instance, so far we did not take into account feedback effects between mechanical stress and wall structural anisotropy during development, as reported in different recent works in cells of the L1 layer [3], [43]–[45]. Although we did not address this level of complexity here, our mechanical model provides a natural framework to model such constitutive laws as the tensor representation of the material properties is naturally adapted to the modeling of local anisotropy.

The morphogenetic model presented in this paper investigates how plant axes and branching systems emerge from basic variables at cellular level (turgor pressure, wall deformation and genetic regulation). This model provides realistic multicellular 3D shape simulations that mainly relies on the presence of a stiff epidermis that is limiting for growth. Remarkably, this is in fact reminiscent of the way a cell wall is limiting the expansion of the protoplast. This similitude was already noticed by Kutschera: “The whole organ can […] be regarded as a single giant cell” [4]. Using realistic 3D mechanical simulations, our computational model supports the view of multicellular axial growth underlying this statement. It shows in addition how lateral organs can be produced by locally regulating the mechanical properties of the epidermis at the tip, while relying on both cell autonomous and non-cell autonomous patterns of tension. Together with Dumais's model on single cell growth [8] also based on anisotropic growth, these models give further support for the existence of a deep self-similarity in the axial growth of plants. This self-similarity is strikingly illustrated by the similitude in the circumferential organization of cortical microtubules within the same tissue, i.e. in a single meristematic cell and in the whole meristem (S1 *A*-*B* Fig.). It is also further supported by the existence of single cell organisms that have developed branching strategies (S1 *C*-*D* Fig.). Caulerpa for instance, a single cell system, exhibits complex tree-like shapes comparable to fern leaves (S1 *E*-*F* Fig.). Altogether, this suggests that plants have found ways through evolution to scale up axial growth in single cells to multicellular systems, using similar biophysical principles. Conversely, this rather supports the idea that growth in plants mainly relies on elongation. In this scenario, cell division in plants would mainly be a way to subdivide an increased volume, with little impact on growth. Such a unifying principle across scales likely reflects the existence of some common essential constraints behind growth. Identifying these core rules, and their exceptions, will be a major challenge for the future of development that only realistic mechanistic models based on the actual effectors of growth will be able to address.

## Models

This section describes the details of our mechanical model of tissue growth. The elastic response being a pure mechanical phenomenon and the growing process a complex biochemical process, we assume that the characteristic elastic-response time (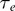) is very small compared to the characteristic time of the growth mechanism (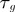). Since our main focus is on the growth process, we will consider time scale 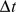 such as: 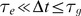.

### Kinematics variables & relations

The positions of material points of 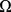 are tracked throughout time. The deformation field 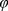 relates the coordinates of a material point in the current, “*observed*” (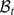) and reference configurations (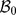) respectively noted 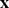 and 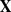: 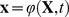
(8)


This field is assumed smooth enough so that its gradient, mapping small variations of length between the material configuration and the current configuration, is defined: 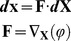
(9)


**Figure 7:**
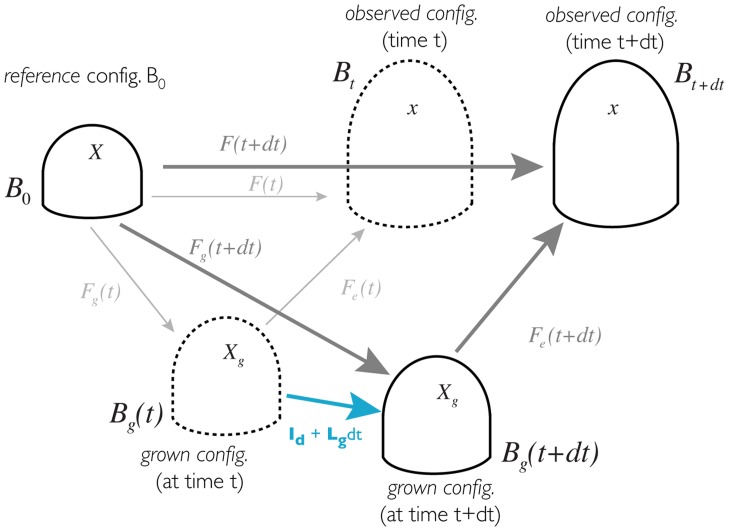
Schematic representation of the different configurations at different time and the deformations between them.

To model growth, the most common approach is to use a multiplicative decomposition of the deformation gradient [22]–[24]

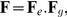
(10)


where the global deformation gradient 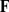 is decomposed into a reversible part 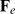 and and irreversible part 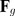. This decomposition leads to an intermediate, “*grown*”, configuration (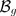), where material coordinates are noted 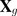. 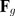 thus maps growth-related deformations between 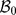 and 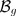 and 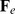 maps purely elastic deformations between 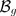 and 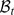, see Fig. 7. In that perspective, growth is regarded as the time evolution of the irreversible part (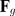) of the deformation gradient field. Geometrically, the time evolution of such deformation gradient fields is estimated through their related velocity gradient fields. In the *grown* configuration 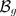, the growth-related part of the velocity gradient field reads as follows:
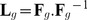
(11)


### Conservation equations

#### Mechanical energy

We assume that the mechanical part of 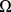 free energy density, noted 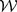, is strictly elastic and only depends on 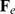, with a local minimum for 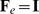. Since it also must be rotation invariant, 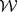 can be expressed as a function of the Green-Lagrangian strain tensor (noted 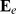) only: 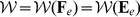
(12)


With the following definition of 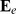:
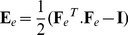
(13)


A straightforward consequence of this assumption is that all the mechanical dissipation processes depend on 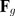 only, which leads to 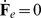 at mechanical equilibrium.

#### Forces balance

As we assume the static equilibrium of the elastic part of the deformation gradient 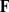, the local formulation of the balance of linear momentum and boundary conditions in the reference ‘grown’ configuration reads: 
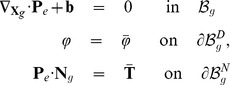
(14)


where 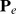, the first Piola-Kirchoff stress tensor, is a measure of the stress applied in the current configuration with respect to the “grown” configuration. 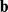 stands for any external force density field and will be neglected hereafter. 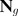 is the unit outward normal to the boundary of the undeformed body. 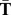 is the prescribed traction per undeformed unit area in part of the boundary 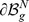. In our case, 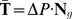 where 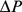 stands for the turgor pressure difference between the inside and the outside of the tissue. Finally, 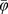 is the prescribed deformation mapping in the rest of the boundaries 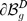 (in our simulations, it corresponds to the fixed basis of the meristem).

### Constitutive equations

To compute the time evolution of the meristem under pressure, we need to define how the cell walls elastically deform and how they grow.

#### Elasticity

We assume a linear strain/stress relationship (Hooke's law): 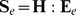 where 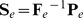 is the second Piola-Kirchoff stress tensor and 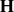 is the Hooke fourth order stiffness tensor. The elastic deformation is thus characterized by a mechanical energy 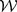:
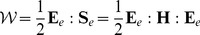
(15)


In its most general form 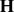 is a fourth order tensor (81 parameters) that relates stresses and strains distributed in three dimensions. For the sake of simplicity, we assume plane stress conditions (since bending forces can be neglected with respect to in-plane forces). Stresses and strains can be described as 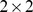 symmetric matrices, downsizing the number of independent coefficients in 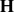 to 6. Using the Voigt notation, the relationship between stress and strain can be written in the following matrix form:
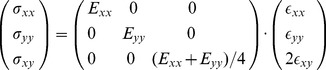
(16)


In order to test our framework with the most simple anisotropic mechanical law possible we chose to neglect the mechanical coupling between directions (null Poisson's ratios in 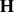), leading to a diagonal form for 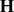 in the Voigt form. The non-null remaining coefficient are the two Young's moduli 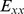 and 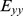 and the shear modulus 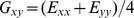. In order to reduce to the bare minimum the number of independent variables in this first set of simulations, we choose a simple expression inspired by the isotropic case.

#### Growth

**Figure 8:**
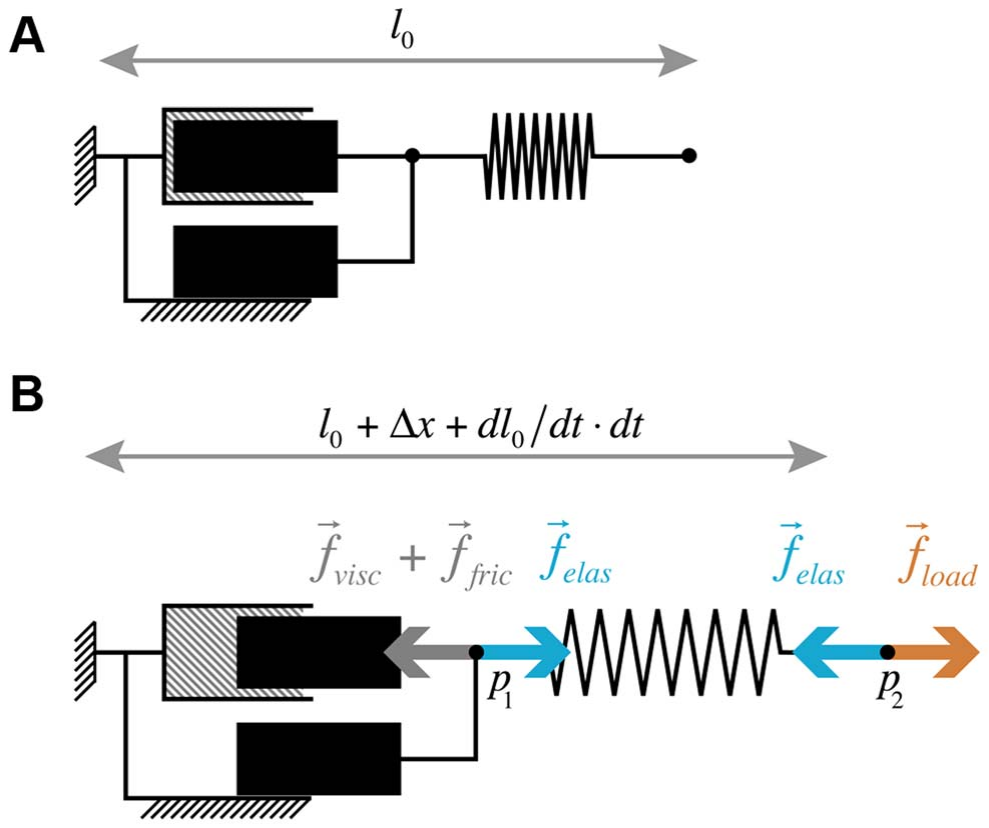
1D version of a unit element of the biomechanical model. a) the system in its resting configuration (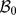). b) the system deformed by a the loading forces, at mechanical equilibrium (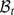). Orange, blue and gray arrows represent respectively the loading (turgor-related) force, the elastic forces and the sum of viscous drag and friction force.

The growth of the wall can be regarded as creep that can be modeled using the Maxwell model of viscoelasticity. In this model, reversible and irreversible phenomena are embodied, respectively, as a purely elastic spring (characterized by an effective spring constant 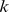) and as a purely viscous dashpot (characterized by an effective viscosity 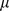), the two being connected in series. Adding a friction force in parallel to the viscous drag enables us to take into account the threshold phenomenon, see Fig. 8. Under loading forces, the mechanical equilibrium of such a system leads to:
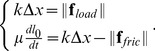
(17)


The first line of Eq.17 refers to the mechanical equilibrium at point 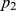 on Fig. 8−*b* and links the elastic stretching of the material to its loading force. The second line refers to the mechanical equilibrium at point 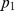 and links the creeping rate of the material to the difference between its elastic stretching and the threshold related force. Using the measure of strain 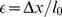 in the system exposed at Eq. 17 leads us to the following expression:
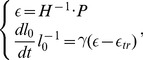
(18)


where we assumed that the friction force is proportional to the initial rest length of the system (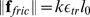) and that the loading force can be expressed as a pressure force 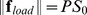 with 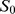 the section on which the pressure 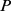 is applied. We also introduced 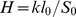 the effective Young's modulus of the material in the considered direction and 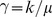 a coefficient characterizing the extensibility of the material in that direction. Extrapolating Eq. 18 in 3D with large deformation leads to the constitutive growth equation:
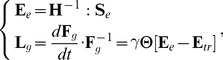
(19)


where 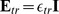 is the threshold matrix strain has to overcome in order to induce growth and 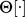 represents the matrix version of the primitive of the Heaviside function, defined as followed:
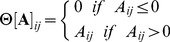
(20)


### Discretization

#### Time discretization

The backbone of the numerical simulation of our model is an iterative loop in which each step represents a time lap 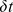 such as: 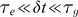
(21)


Each time step happens as follow:

1. at the beginning of the 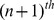 step the system is already at mechanical equilibrium, deformed by the loading forces. If the strain tensor 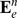 is above the threshold value 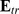 growth is initiated.

2. Once growth has been initiated, the growth-related deformation 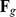 is updated from its current value 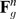 to a new one 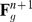 established by Eq. 25.

3. Once 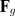 has been updated, mechanical equilibrium is computed and the strain tensor is updated with value 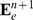. A new step can begin.

#### Incremental evolution of 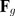


By discretizing time in steps of duration 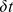, we can express deformation 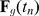 (with 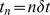) as the multiplication of incremental deformations 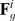. Moreover, deformation at time 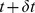 is directly related to deformation at time 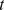:
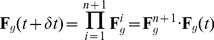
(22)


Since 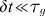, the instantaneous growth rate (noted thereafter 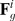) is supposed constant over each step. Therefore each element of the multiplicative decomposition (Eq. 22) can be expressed in terms of its corresponding instantaneous growth rate: 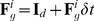
(23)


Applying forward finite differences to 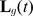 leads to: 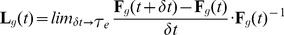
(24)


Combining Eqs.22),(23, 24 & 19 enables us to estimate the 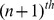 growth-related deformation 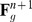 knowing the strain tensor at step 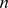:
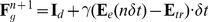
(25)


#### Space discretization

We have chosen to discretize the continuous model of cell walls using first order finite elements. Nodes are placed at each wall junction, and triangles obtained by tessellation. Material points are interpolated from nodes, based on linear (barycentric) shape functions 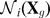: 
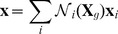
(26)


where 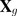 are 2-dimensional material coordinates in the ‘grown’ configuration. For simplicity, we match material principal directions with mechanical anisotropy. By spatial differentiation, we obtain 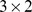 elastic deformation gradient matrices, which are uniform over each triangle: 
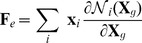
(27)


Nodal elastic forces in the current configuration are spatially integrated using midpoint quadrature. One evaluation of the energy density per element is sufficient since it is uniform within each triangle: 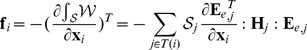
(28)


where 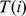 are the adjacent triangles of node 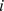. 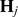, 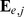 and 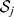 are the Hooke tensor, strain tensor and surface of triangle 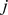 (‘grown’ configuration).

For inner cell walls, where no information of mechanical anisotropy is available, we assume that the representation of mechanical properties can be simplified by using one dimensional elasto-plastic elements - or springs- between cell vertices.
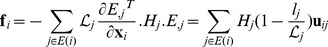
(29)


where 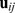 is a unit vector of edge 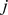 in the direction of node 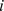, 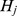 is the spring stiffness and 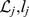 the ‘grown’ and current length of edge 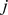.

Turgor pressure is converted into nodal external forces by spatial integration: 
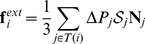
(30)


where 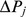 is the pressure (supposed uniform) on triangle 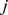. 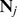 is the outward unit normal (current configuration).

#### Time evolution

Solving the weak form of Eq. 14, turns out to minimize the total elastic. We solve this by gradient descent: 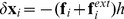
(31)


where 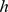 is the step size. For faster convergence and stability, we use an implicit scheme. At iteration 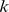, we have: 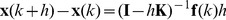
(32)


where 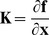 is the stiffness matrix. 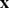 and 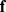 are the position and force vectors of all nodes (concatenation of the 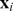 and 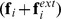). This linear system is solved using the conjugate gradient algorithm.

#### Computational simulation framework

We have implemented our mechanical model in the open source software SOFA [39]. Its modularity allowed us to combine different element types (triangle and edge elements), forces (elastic forces and turgor) and positional constraints within the same model. At each step of growth, the software is used to find the static elastic equilibrium, Eq. 14 given a current configuration. The use of an implicit integration scheme makes it possible to achieve close-to interactive simulation of growth.

## Supporting Information

S1 Fig **Axial growth self-similarity in plants.** (A). NPA-grown seedling exhibiting a naked SAM expressing the GFP-MBD construct. (B) 93 h after microtubule depolymerization, a meristematic cell expressing the GFP-MBD construct has grown without dividing, hence its increased size, and has repolymerized its microtubules. Note the presence of circumferential microtubule orientations at the periphery and random microtubule orientations in the center in both A and B. (C) Longitudinal section through an Arabidopsis SAM (From [46]). (D) Longitudinal section through the phylloid growing tip of unicellular algae Caulerpa taxifolia (Adapted from [47]). Note the morphological similarities between C and D. (E) Drawing of the common fern Polypodium vulgare, highlighting its rhizome and composite frond (From [48]). (F) Picture of unicellular green algae Caulerpa taxifolia, highlighting its creeping cauloid and composite phylloid (Adapted from [47]). Note the similarities in architectures.
(TIF)

S1 Text **Supporting information.** Units and parameter values used in simulations corresponding to Fig. 4, Fig. 5, and Fig. 6.
(PDF)

S2 Text **Software installation.** This text describes the procedure to install our software and to run the mechanical model.
(DOCX)

S1 Movie **Growth of a dome of homogeneous cells.** All cells are isotropic with identical elasticity, plasticity threshold and growth speed. See also Fig. 4.B.
(MP4)

S2 Movie **Axial growth.** Mechanical anisotropy is imposed to the bottom cells in the epidermis to model the effect of microtubules orientation. The selected plasticity threshold permits axial growth only and restrains radial growth. See also Fig. 4.C.
(MP4)

S3 Movie **Imposing anisotropy to 80% of the dome height.** Red cells are anisotropic to model alignment of microtubules orientation while blue cells are isotropic. The growth of the dome produces an axial shape. See also Fig. 4.D.
(MP4)

S4 Movie **Imposing anisotropy to 40% of the dome height.** Red cells are anisotropic to model alignment of microtubules orientation while blue cells are isotropic. The growth of the dome produces a globular shape. See also Fig. 4.D.
(MP4)

S5 Movie **Growth with a gradient of anisotropy.** The bottom cells have maximum anisotropy while top cells are perfectly isotropic. See also Fig. 4.E.
(MP4)

S6 Movie **Creation of a lateral dome by decreasing cell wall rigidity in a primordium region.** The frontier between the main axis and the lateral bump is not well marked. See also Fig. 4.F.
(MP4)

S7 Movie **Non-cell autonomous growth where rigidity of cells in the inner layers has been decreased by a 10-fold factor.** No bump emerges. See also Fig. 4.G left.
(MP4)

S8 Movie **Transversal cut of the simulation of** **Fig. 4** **.F.** See also Fig. 4.G middle.
(MP4)

S9 Movie **Non-cell autonomous growth where turgidity of cells in the inner layers has been increased by a 2.5-fold factor.** Only a shallow bump tends to emerge. See also Fig. 4.G right.
(MP4)

S10 Movie **Creation of a lateral dome with a marked frontier by increasing cell wall rigidity in the cells surrounding the primordium.** See also Fig. 4.H.
(MP4)

S11 Movie **Creation of a lateral dome with a marked frontier by introducing anisotropy in the frontier region.** The cell wall rigidity in the cells surrounding the primordium is made stiffer in the circumferential direction only. See also Fig. 4.H.
(MP4)

S12 Movie **Increasing growth rate in the primordium to facilitate the emergence of a lateral dome.** Compared to simulation of Fig. 4.I., the necessary decrease of rigidity of the cell wall in the primordium is less important and is compensated by the increase of growth rate. See also Fig. 4.J.
(MP4)

S13 Movie **Initiating a asymmetric lateral dome.** Frontier region is only limited to the top part of the primordium. Even with no frontier at the bottom, a globular dome emerges normal to the surface. See also Fig. 5.J-K.
(MP4)

S14 Movie **Tentative creation of an asymmetric lateral dome with stiffer adaxial region.** Primordium region is subdivided into abaxial and adaxial regions. With stiffer adaxial cells, upward development of the primordium is limited. See also Fig. 5.L-M.
(MP4)

S15 Movie **Tentative creation of an asymmetric lateral dome with stiffer abaxial cells.** Upward development of the primordium is predominant. See also Fig. 5.N-O.
(MP4)

S16 Movie **Creation of an asymmetric lateral dome.** Abaxial cells are made stiffer and anisotropic. See also Fig. 5.P-Q.
(MP4)

S17 Movie **Mechanical simulation of a flower bud with outgrowth of sepal primordia.** Four regions corresponding to the sepal primordia are defined with a frontier region that surrounds the primordia. Each region is given specific wall stiffness, anisotropy and growth speed corresponding to different gene expression. See also Fig. 6.
(MP4)

S18 Movie **Characterization of residual stress after removal of the turgor pressure.** The simulation of Fig. 4.I is used as starting point with its turgor pressure removed. The stress of some regions shows incompatibilities of rest positions of neighbor elements.
(MP4)
