# Correction: A Computational Framework for 3D Mechanical Modeling of Plant Morphogenesis with Cellular Resolution

**DOI:** 10.1371/journal.pcbi.1005027

**Published:** 2016-07-08

**Authors:** The PLOS Computational Biology Staff

There are some errors in Equations 11 and 20.

Equation 11 should be replaced by the following:

$$L_{g}={\dot{F}}_{g}\cdot F_{g}^{-1}$$

Equation 20 should be replaced by the following:

$$\Theta (A)=P_{A}\theta (P_{A}{}^{T}AP_{A})P_{A}{}^{T}$$

where **P**_**A**_ is the change-of-basis matrix from **A** to the basis of eigenvectors of tensor **A** and:

$$\theta {\left[A\right]}_{ij}=\{\begin{array}{ccc} 0 & if & A_{ij}\leq 0\\ A_{ij} & if & A_{ij}>0 \end{array}$$

The authors confirm that the errors do not affect the results of the paper.
