# Supplementary figures and images for: A Computational Framework for 3D Mechanical Modeling of Plant Morphogenesis with Cellular Resolution
Source: PLoS Comput Biol. 2015 Jan 8;11(1):e1003950. doi: 10.1371/journal.pcbi.1003950 (PMC4288716; doi:10.1371/journal.pcbi.1003950)

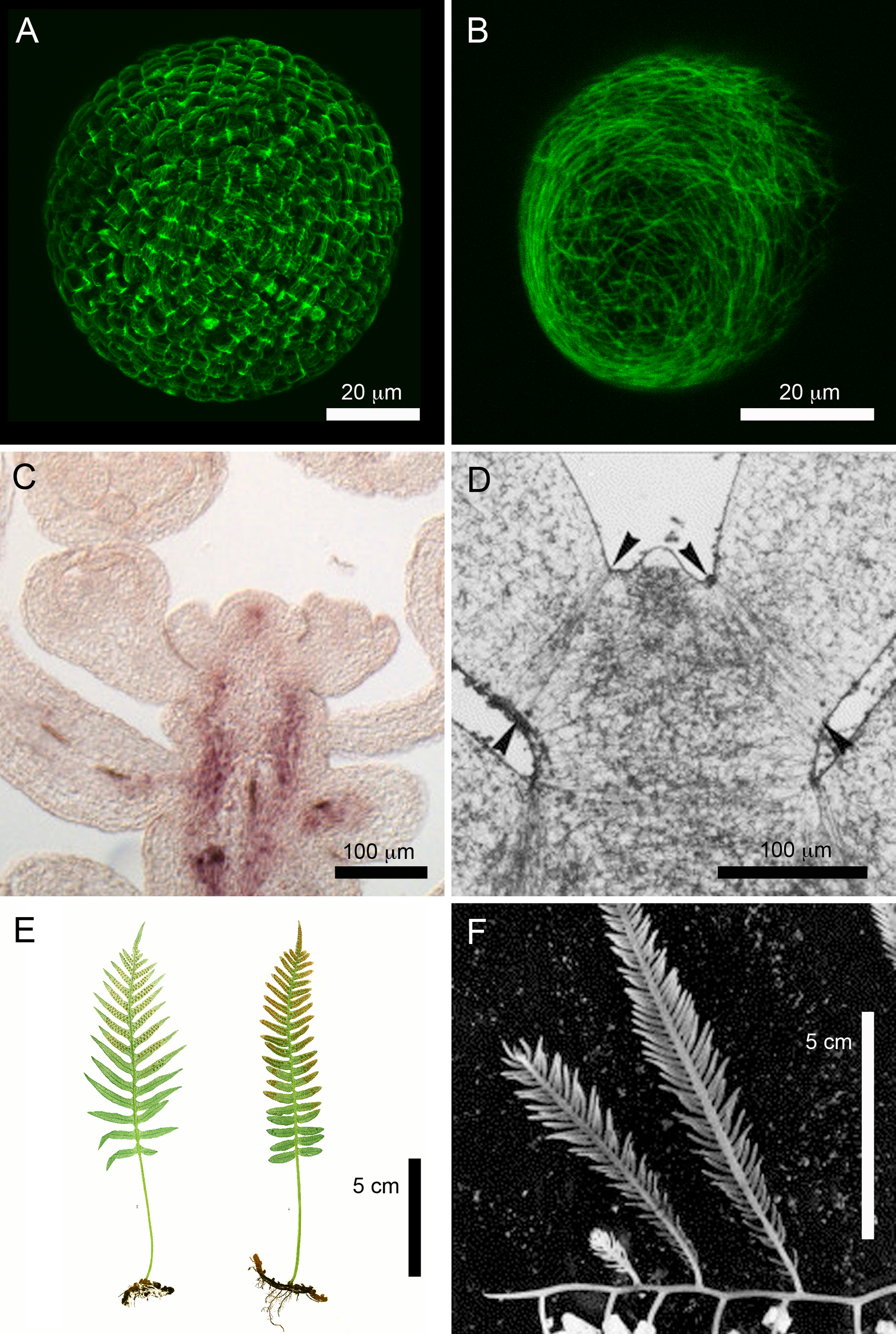

Supplement: S1 Fig — Axial growth self-similarity in plants. (A). NPA-grown seedling exhibiting a naked SAM expressing the GFP-MBD construct. (B) 93 h after microtubule depolymerization, a meristematic cell expressing the GFP-MBD construct has grown without dividing, hence its increased size, and has repolymerized its microtubules. Note the presence of circumferential microtubule orientations at the periphery and random microtubule orientations in the center in both A and B. (C) Longitudinal section through an Arabidopsis SAM (From [46]). (D) Longitudinal section through the phylloid growing tip of unicellular algae Caulerpa taxifolia (Adapted from [47]). Note the morphological similarities between C and D. (E) Drawing of the common fern Polypodium vulgare, highlighting its rhizome and composite frond (From [48]). (F) Picture of unicellular green algae Caulerpa taxifolia, highlighting its creeping cauloid and composite phylloid (Adapted from [47]). Note the similarities in architectures. (TIF) [file pcbi.1003950.s001.tif]
