# Supplementary material for: A Computational Framework for 3D Mechanical Modeling of Plant Morphogenesis with Cellular Resolution
Source: PLoS Comput Biol. 2015 Jan 8;11(1):e1003950. doi: 10.1371/journal.pcbi.1003950 (PMC4288716; doi:10.1371/journal.pcbi.1003950)
Supplement: S1 Text — Supporting information. Units and parameter values used in simulations corresponding to Fig. 4, Fig. 5, and Fig. 6. (PDF) [file pcbi.1003950.s002.pdf]

Supporting Information

F. Boudon et al.

Parameter units and values used in the simulations

All the simulations assume a turgor pressure of $0.4MPa$ (except specified otherwise). For the triangle finite elements, the first material direction is oriented with the first direction of rigidity (characterized by the effective Young's modulus E_{xx}) following circumpherentially the meristem dome and a second direction (with the effective Young's modulus E_{yy}) set vertical. The effective shear modulus G_{xy} is assumed to be equal to $(E_{xx}+E_{yy})/4$. In our simulations, Young's modulus of triangle finite elements are scaled according to the thickness (assumed roughly equal to 300 nm in the epidermis and 60 nm inside). And therefore, all the effective Young's modulus values used in our simulations and given in the various tables thereafter are in $MPa \cdot \mu m$. The first simulation has the same parameters for all elements.

Simulation	E_{xx}	E_{yy}	$\mathbf{E}_{tr}$
4.B	400	400	0.0

The simulation 4.C uses a gradient of anisotropy (noted AG). Top horizontal cells are isotropes while bottom vertical cells have the highest degree of anisotropy. Anisotropy of intermediate cells are determine from their initial orientations. In simulation 4.D, a top anisotropic region and a isotropic bottom region are defined.

Simulation	TOP		BOTTOM			
E	E_{xx}	E_{yy}	E_{xx}	E_{yy}	$\mathbf{E}_{tr}$	AG
4.C	750	750	1200	300	0.25	Yes
4.D	750	750	1200	300	0.25	No
4.E	750	750	1200	300	0.005	Yes

All the next simulations assume a strain threshold $\mathbf{E}_{tr}$ of 0.005 and use a gradient of anisotropy. In the next simulation, three cells regions are defined : the primordium (PRIM), the central zone (CZ) and the periphery (PERI). Finite elements of the cells of these regions are defined with specific parameters.

	PERI		PRIM		CZ	
E	E_{xx}	E_{yy}	E_{xx}	E_{yy}	E_{xx}	E_{yy}
4.F	1200	300	125	125	750	750

In the simulation 4.G, influence of internal tissues is tested. For this an internal primordia region (IPR) and an internal meristem region (IMR) (i.e. the rest of the internal cells) are defined. By default the rigidity of the epidermis tissue are defined in the following way.

	PERI		PRIM		CZ	
E	E_{xx}	E_{yy}	E_{xx}	E_{yy}	E_{xx}	E_{yy}
4.G	1200	300	1200	300	750	750

In simulation 4.G.left, the decrease of rigidity in internal tissue of the primordia is tested. The young modulus of the internal cells are defined with the values given in the next table. Note that these values take into account the difference of thickness with the epidermis.

	IMR		IPR	
E	E_{xx}	E_{yy}	E_{xx}	E_{yy}
4.G.left	80	80	8	8

In simulation 4.G.right, the increment of pressure in internal tissue of the primordia is tested using the following parameters.

P	IMR	IPR
4.G.right	0.4	1

For the next simulations, an additionnal frontier (FRT) region around the primoridum is defined with specific orientation: the first direction (corresponding to E_{xx}) is defined tangential to the contour of the primordium and the second one (E_{yy}) toward the primordium.

	PERI		PRIM		CZ		FRT	
E	E_{xx}	E_{yy}	E_{xx}	E_{yy}	E_{xx}	E_{yy}	E_{xx}	E_{yy}
4.H	1200	300	125	125	750	750	1200	1200
4.I	1200	300	125	125	750	750	1600	125
4.J	1200	300	250	250	750	750	1600	125

The simulation 4.J is exploring the influence of growth rate. Various growth rates ($\gamma \propto [T]^{-1}$), given in arbitrary unit) are assigned to the different zones.

γ	PERI	PRIM	CZ	FRT
4.H	0.5	1.0	0.5	0.5

In the series of simulation corresponding to Fig. 5, the primordium is subdivided into abaxial (ABP) and adaxial (ADP) sub-regions. The frontier region is reduced only to the upper half contour of the primordium. The periphery, the central zone and the frontier regions have the following parameters for all the simulations.

	PERI		CZ		FRT	
E	E_{xx}	E_{yy}	E_{xx}	E_{yy}	E_{xx}	E_{yy}
5	1200	300	400	400	1000	200

The primordium regions have specific parameters values, given in the following table, for the various simulations.

	ABP		ADP	
E	E_{xx}	E_{yy}	E_{xx}	E_{yy}
5.J-K	120	120	120	120
5.L-M	120	120	300	300
5.N-O	300	300	120	120
5.P-Q	300	120	120	120

Finally, the last simulation displays four different primordium regions (subdivided themselves into abaxial and adaxial sub regions). The first primordium region corresponds to the adaxial side of the meristem, the second to the abaxial side and the two lasts to the lateral sides. The parameters values used in this simulation are recap in the following table:

Zone	E_{xx}	E_{yy}	γ
CZ	400	400	0.2
PERI	1200	240	0.2
FRT	4000	200	0.2
ADP1	120	120	1
ADP2	120	120	0.7
ADP3-4	120	120	0.5
ABP1	600	160	1
ABP2	600	160	0.7
ABP3-4	600	160	0.5
