# Supplementary material for: A Computational Framework for 3D Mechanical Modeling of Plant Morphogenesis with Cellular Resolution
Source: PLoS Comput Biol. 2015 Jan 8;11(1):e1003950. doi: 10.1371/journal.pcbi.1003950 (PMC4288716; doi:10.1371/journal.pcbi.1003950)
Supplement: S2 Text — Software installation. This text describes the procedure to install our software and to run the mechanical model. (DOCX) [file pcbi.1003950.s003.docx]

Installation

The computational framework described in the manuscript is built upon two software platforms, namely Sofa and [OpenAlea](http://openalea.gforge.inria.fr/). The first one provides numerical tools for physical simulation. The second contains data structure and algorithm to manipulate 3D structure of vegetal tissue. An extra software module, named sofatissue, makes the link between the two platforms and defines the different scenario.

These software modules are coded using the programming languages C++ and Python and rely on a number of third party libraries (such as Qt, OpenGL, libQGLViewer, Boost, CGAL). They were written to be compilable and executable on multiple operating systems (Windows, Linux and Mac Os X). Installation instructions or installer for each platforms are given on their website for each operating systems. However, we currently focus our developments on the Mac Os X system and gives more precise installation instructions to build up the framework at the end of this document.

## Installation procedure

- Install OpenAlea. (See its [*Download* page](http://openalea.gforge.inria.fr/dokuwiki/doku.php?id=download)). Once installed, the Python modelling language and a number of scientific software modules will be available. To test if the installation succeeded, you can try to launch *visualea* to start the visual programming editor of the platform.
- Install Sofa. (See its [*Getting Started* page](http://wiki.sofa-framework.org/wiki/Getting_Started)). To test if the installation succeeded, you can launch *runSofa*.
- Get the sofatissue module and unzip it.

wget <https://gforge.inria.fr/frs/download.php/file/33843/sofatissue.tgz>

tar xzf sofatissue.tgz

## Playing simulation

To test a simulation, you should go in the scenario directory of the sofatissue module.

$ cd sofatissue/scenario

To test simulation of figure 6 for instance, you can type the following command line.

$ runSofa flowerbud.py

To give more details on the sofatissue module, it is organized in the following way.

It contains three main directories: data, meca and scenario. *data* contains various definition of vegetal tissue; *meca* contains source code to manage a simulation and *scenario* the definition of the parameters values of the simulations.

## Detailed installation procedure on Mac Os X 10.9

Installing dependencies of Sofa and OpenAlea (Inspired from page <http://openalea.gforge.inria.fr/dokuwiki/doku.php?id=documentation:user:installation_mac>)

- Install [MacPort](https://www.macports.org/install.php)
  - For this, install [Xcode](https://developer.apple.com/technologies/tools/) and [XQuartz](http://xquartz.macosforge.org/)
- Install the following software modules

# Update MacPort

sudo port -v selfupdate

# Install Python 2.7

sudo port install python27

# Make python2.7 the default

sudo port select --set python python27

# Install the Scientific Python stack

sudo port install py27-numpy py27-scipy

# Install Qt and PyQt

sudo port install qt4-mac

sudo port install py27-pyqt4 +scintilla

sudo port install py27-qscintilla

sudo port install py27-matplotlib -tkinter +qt4 +webagg

# Install other Python utilities

sudo port install py27-pil py27-opengl py27-nose py27-sphinx

sudo port select --set sphinx py27-sphinx

sudo port install py27-ipython +notebook +parallel +pyqt4

# Make ipython2.7 the default

sudo port select --set ipython ipython27

# Install utilities

sudo port install subversion

# Install C++ libraries

sudo port install scons qhull

sudo port install gnuplot -aquaterm -luaterm -pangocairo -wxwidgets +qt sudo port install boost bison flex

sudo port install cgal libANN

sudo port install py27-PyQGLViewer

sudo port install cmake +gui

- Install [OpenAlea from sources](http://openalea.gforge.inria.fr/dokuwiki/doku.php?id=download:source_distribution#building_and_installing_for_the_user_recommended2)

# Getting the sources

svn checkout <https://scm.gforge.inria.fr/svn/openalea/trunk> openalea

wget <https://gforge.inria.fr/frs/download.php/file/33842/vplants-for-sofa.tgz>

tar xzf vplants-for-sofa.tgz

wget <https://gforge.inria.fr/frs/download.php/file/33843/sofatissue.tgz>

tar xzf sofatissue.tgz

# Compiling the sources

cd openalea

python multisetup.py install

cd ../vplants-for-sofa

python multisetup.py install

- Install Sofa from source

# Getting the sources

git clone git://scm.gforge.inria.fr/sofa/sofa.git

# Configure Sofa source to use Python from macport

cd sofa/applications/plugins/SofaPython/

# edit CMakeLists.txt and comment lines 11 and 12.

# Configure Sofa compilation

cd sofa

cmake-gui .

# run the configure button

# In Sofa-Plugin, enabled options SOFA-PLUGIN_SOFAPYTHON and SOFA-PLUGIN_FLEXIBLE

# run the configure and then generate buttons.

# Compile

make
